# Nephron-Sparing Surgery for Renal Masses Measuring Larger Than 7 cm on Preoperative Imaging: A Single Surgeon, Single Center Experience

**DOI:** 10.1155/2013/691080

**Published:** 2013-04-04

**Authors:** Tarık Esen, Ömer Acar, Ahmet Musaoğlu, Metin Vural, Sergin Akpek

**Affiliations:** ^1^School of Medicine, Koc University, Istanbul 34450, Turkey; ^2^Department of Urology, VKF American Hospital, Istanbul 34365, Turkey; ^3^Department of Radiology, VKF American Hospital, Istanbul 34365, Turkey

## Abstract

*Objectives*. To document the feasibility of nephron-sparing surgery (NSS) for the surgical treatment of renal masses measuring larger than 7 cm (cT2) on preoperative imaging. *Methods*. A total of 139 patients have undergone NSS between 2001 and 2012 by a single surgeon in our clinic. Of these, we identified 17 patients whose tumors were measuring greater than 7 cm on preoperative imaging studies and were limited to the kidney. Their charts were retrospectively reviewed. *Results*. Mean age of the study population was 49.8 ± 11.3 years. Thirteen patients were managed by open NSS, while 4 patients have undergone robot-assisted NSS. Mean diameter and mean R.E.N.A.L. score of the tumors that were enucleoresected were 8.2 cm and 8.5, respectively. A total of 5 Clavien grade 2 and higher complications were recorded within 30 days of surgery. Histopathologic examination revealed benign histology in almost 1/4 of the cases. After a median followup of 33 months, all of our patients were alive. Only one patient (5.8%) experienced local recurrence. *Conclusions*. NSS is a feasible and safe option for large (>7 cm) renal masses. It may be considered not only for imperative conditions but also for highly selected cases with a normal contralateral kidney.

## 1. Introduction

Nephron-sparing surgery (NSS) offers equivalent oncologic control, as does radical nephrectomy in patients with appropriately selected stage T1 renal tumors [1, 2]. Additionally, NSS has been associated with decreased risks for renal impairment [3–5] and cardiovascular events [6, 7], which may explain the improved overall survival, documented in partial nephrectomy series [8]. Current guidelines emphasize NSS for all anatomically amenable T1 renal tumors [9].

As the benefits of NSS become apparent, the indications may continue to expand [10]. In order to establish standards for future comparisons, we must fully appreciate the efficacy, safety, and limitations of NSS for renal masses larger than 7 cm. Therefore, we reviewed our experience and reported the results in 17 patients with renal masses, which had a maximum diameter of more than 7 cm on preoperative imaging studies, treated with either open or robot-assisted laparoscopic NSS during 13 years.

## 2. Methods

Between 2001 and 2012, a single surgeon (TE) performed 139 NSSs (97 open, 35 robot-assisted, and 7 laparoscopic NSSs) in one center. We retrospectively reviewed the charts of the patients, whose renal tumors measured more than 7 cm in diameter on preoperative imaging (*n* = 17, 17.52%). Preoperative evaluation consisted of laboratory tests and cross-sectional imaging studies (computerized tomography and/or magnetic resonance imaging). Morphometric characteristics of the renal masses were assessed by R.E.N.A.L. (radius, exophytic/endophytic, nearness, anterior/posterior, location) scoring system [11]. Based on preoperative radiologic findings, none of the patients had lymph node involvement or distant metastasis.

Open nephron-sparing surgery (ONSS) was performed using the intercostal (between 11th and 12th ribs) extraperitoneal flank approach, as previously described elsewhere in detail [12]. Robot-assisted nephron-sparing surgeries (RANSSs) were performed using the da Vinci surgical system (Intuitive Surgical, Inc., Sunnyvale, CA, USA) with a 5-port approach, including two 8 mm ports for robotic instruments, one 12 mm port for the robotic scope, and 2 ports for the bedside assistant. RANSSs were carried out through the transperitoneal route with the patient in the flank position. After demarcating tumor margins with electrocautery, enucleoresection was carried out using cold scissors, leaving a minimal margin of normal parenchyma [13]. The tumor bed was overseen with 3–0 polyglactin sutures (in case of pelvicalyceal violation), and parenchyma was adapted with the sliding clip technique. Argon laser coagulation or hemostatic materials were used as needed. Operative data consisted of total operative time, estimated blood loss (EBL), warm-ischemia time (WIT), and adverse events.

Technical, renal functional, and oncological outcomes were abstracted from our database. All complications within 30 days of surgery were classified according to the Clavien-Dindo classification system [14]. Estimated glomerular filtration rate (eGFR) was determined before and after NSS using the modified Modification of Diet in Renal Disease (MDRD) equation [15]. Chronic kidney disease stage was assigned according to the National Kidney Foundation definition [16]. Pathological data included histological subtype, grade, and margin status. Tumor staging was designated according to the TNM classification based on the 2009 American Joint Committee on Cancer/International Union Against Cancer classification system. Tumor size was defined as the largest diameter of the tumor (cm). Tumor recurrence was defined as a new renal mass in the resection bed of the kidney in the absence of distant metastasis based on imaging. Followup was calculated from the time of surgery until the last known contact with the patient or until the date of death. Mortality status was confirmed with death certificates.

Statistical calculations were performed using the commercially available software (SPSS version 20). Student's *t*-test and Fisher's exact test were the statistical methods of reference.

## 3. Results

**Figure 1:**
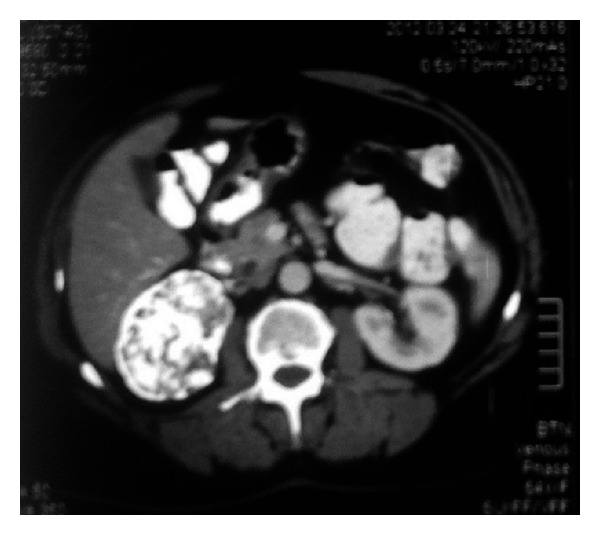
A 49-year-old female patient who had a cT2 mass in her right kidney. She was operated through the open route under warm ischemia. Final pathologic diagnosis was Fuhrman grade 2, clear cell RCC, pT2a.

**Figure 2:**
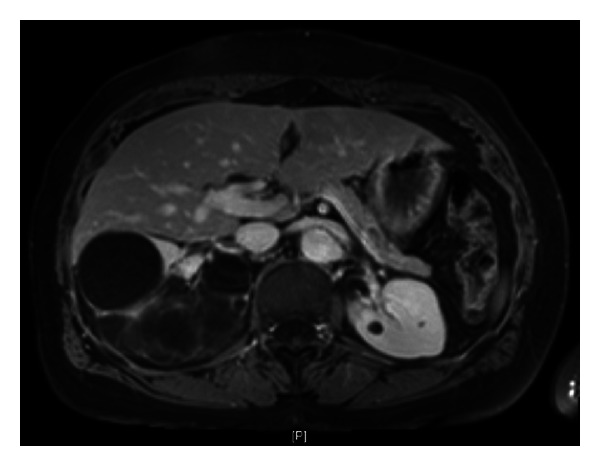
A 66-year-old female with a multiloculated cystic mass measuring 14.5 cm in its greatest dimension. This right renal mass harbored thin septa formations that enhanced after contrast administration. She was managed with robot-assisted NSS without hilar clamping. Final pathologic diagnosis was cystic nephroma.

A total of 17 patients with clinical T2 renal masses (>7 cm) have undergone nephron-sparing surgery between 2001 and 2012, at our institution. Thirteen patients (76.4%) were treated through the open route (Figure 1), while 4 patients (23.5%) were managed by RANSS (Figure 2). Mean age of the study population was 49.8 ± 11.3 years (range = 30–66). Male to female ratio was 1.42 (10/7). Mean ASA score of the operated patients was 1.1 ± 0.39 (range = 1–3). The majority of the tumors were discovered incidentally (*n* = 11, 64.7%). NSS was offered based more commonly on elective indications. The indication to perform NSS was imperative (bilateral mass *n* = 2, solitary kidney *n* = 1) in 3 patients (17.6%).

Mean tumor size was 8.24 ± 2.45 (range = 6–15) cm according to the final pathology reports. Diameter of the resected tumor was measured less than 7 cm in 5 patients (29.4%) whose preoperative imaging findings were suggestive of a T2 lesion. Mean tumor size in these 5 cases was 6.1 ± 0.14 (range = 6–6.3). Mean R.E.N.A.L. score was 8.5 (range = 6–10).

**Figure 3:**
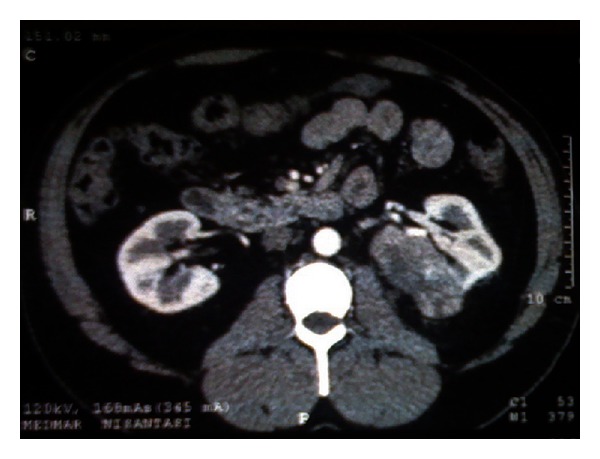
A 51-year-old male patient with a cT2 mass located on the posteromedial aspect of the left kidney. His operation was initiated in a minimally invasive fashion. However, open conversion was necessary because of the size and posterior location of the tumor. Final pathologic diagnosis was Fuhrman grade 2, clear cell renal cell carcinoma, pT2a.

Mean operative duration and mean estimated blood loss amount were 125 ± 35.4 minutes (range = 75–220) and 267.6 ± 117.1 mL (range = 100–500), respectively. Mean warm-ischemia time was 15.2 ± 4.1 minutes (range = 10–21) in the 8 surgeries (47%) during which tumor was enucleoresected after clamping the renal pedicle. The difference between the mean R.E.N.A.L. scores of the tumors that were managed under ischemic and nonischemic conditions was statistically insignificant (8.6 versus 8.4  *P* = 0.72). Open conversion during robot-assisted surgery was necessary in 2 cases, whose tumors had R.E.N.A.L. scores of 9 and 10, respectively (Figure 3).

**Figure 4:**
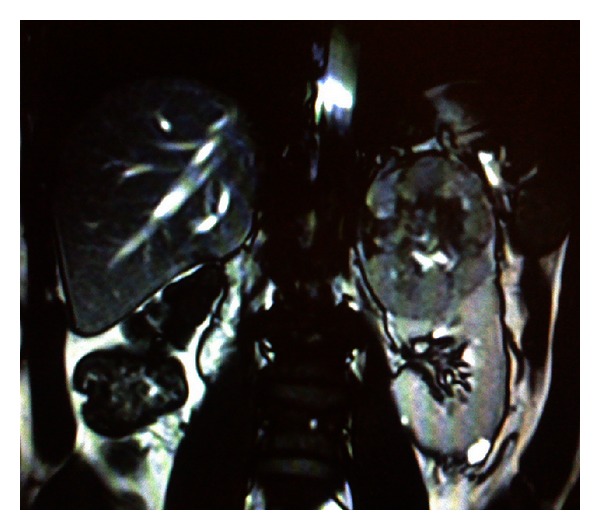
A 59-year-old male patient who had undergone right radical nephrectomy due to renal cell carcinoma, 16 years ago. He was operated due to a cT2 mass originating from the upper pole of his solitary kidney. The mass was excised through the open route, under perfused conditions.

Pathologic information has been summarized in Table 1. According to the final pathology reports, surgical margins did not harbor malignant infiltration in any case. Patients were hospitalized for a mean duration of 5 ± 5.4 days (range  =  3–26). Three patients suffered from a total of 6 grade 2 and higher complications within 30 days of surgery. Blood transfusion and bladder recatheterization due to gross hematuria were the recorded grade 2 and 3a complications in 2 patients, respectively. Blood transfusions, angioembolization, double-j catheter insertion due to urinary extravasation, and temporary hemodialysis are counted for the grade 2, 3a, 3b, and 4a complications encountered during the postoperative course of another patient who had a 9 cm enhancing mass in his solitary kidney (Figure 4).

Mean preoperative and postoperative eGFR was 89.3 ± 20.5 mL/min/1.73 m^2^ (range = 60–136.1) and 76.9 ± 23.5 mL/min/1.73 m^2^ (range = 8.3–111.6), respectively. The difference between these 2 mean values was statistically insignificant (*P* = 0.11).

Mean duration of follow-up was 35.2 ± 28.1 months. During the followup period, tumor bed recurrence was detected in one patient (5.8%), who underwent radical nephrectomy as a complementary procedure in another center. None of the patients in this cohort was lost either due to RCC or due to other comorbidities.

## 4. Discussion

Multiple previous studies have shown the oncological equivalency of NSS to radical nephrectomy (RN) for T1a and even T1b renal masses [2, 24–27]. Considering the facts that some of these localized renal lesions will be benign and/or low grade, and renal preservation has documented benefits on long-term cardiovascular health [6–8], the guidelines advocate NSS as a standard of care for all T1 renal masses [9].

Although, there is a paucity of clinical series evaluating the oncologic results of NSS for T2 renal lesions, initial results are encouraging (Table 2). Breau et al. found that cancer-specific survival and overall survival were similar between NSS and RN for T2 tumors or greater [20]. Karellas et al. found that 71% of the patients were alive and free of disease at a median followup of 17 months [19]. Becker et al. reported 5-year overall and cancer-specific survival rates of 88.0% and 97.0%, respectively [18]. Conversely, Hafez et al. found a significant increase in recurrence rates as well as decreased survival in their study consisting of partial resections performed for tumors ≥ 7 cm in size [23]. It is also important to note that a recent analysis of almost 19.000 localized RCCs in the SEER database showed that greater than 75% of localized renal cancers > 7 cm in size and more than 65% of those >10 cm in size are low-grade lesions, suggesting that NSS for large renal masses may be oncologically sound [28]. In our series, after a median followup of 33 months, all 17 patients were alive and free of disease. Local recurrence was detected only in one patient (5.8%), 10 months after the nephron-sparing procedure.

Undoubtedly, NSS for tumors larger than 7 cm represents a surgical challenge, especially in relatively unexperienced hands. The surgical team has to decide upon the route (open, laparoscopic, or robot assisted NSSs) by which resection is going to be carried out. The idea and the method of (clamping the vasculature or manual parenchymal compression) creating ischemic conditions is another tough decision to be made. Maintaining hemostasis and reconstructing the pelvicalyceal system over a large parenchymal defect might be technically demanding especially while performing minimally invasive NSS. Surgical expertise, technologic availabilities, and patient preference will definitely have an influence on the surgical choices.

Recently described morphometric scoring systems may be used to predict operative complexity. Hence, preoperative morphometric data might tailor the surgical approach. In our series, mean R.E.N.A.L. score of the tumors that were resected under perfused conditions was insignificantly lower than that of the tumors operated under ischemic conditions. Although being lower in the robotic group, the difference between robot-assisted group (*n* = 4) and open surgical group (*n* = 13) in terms of mean R.E.N.A.L. score was also statistically insignificant (8.2 versus 8.6, *P* = 0.58). However, these differences might gain statistical significance as the numbers increase. Furthermore, none of the patients for whom open surgery was employed had a tumor with low R.E.N.A.L. score (4–6). Conversely, one patient (25%) in the robotic group had a tumor in the low R.E.N.A.L. score category.

Postoperative period may also be challenging after NSS for T2 tumors. Long et al. evaluated the results of 46 patients with 49 clinical T2 renal tumors and reported a major urological complication in the form of a urinary fistula in six (12.2%) patients [17]. In the other reported series for cT2 NSSs, the rate of urinary fistula was in the range of 3.3 to 18.8% [18–23]. In our series, only one patient (5.8%) suffered from urinary extravasation. Furthermore, our transfusion rate was 11.7% (*n* = 2), which is concordant with the range reported in the literature (0–30%).

As shown by previous studies, almost 1/3 of the clinical T2 tumors were either benign or represented a very low-grade malignancy (chromophobe RCC), emphasizing the importance of renal preservation when technically feasible, regardless of tumor size [17, 18]. In our cohort, 23.5% of the tumors were reported to have benign histology, while 46% of the RCCs were of either papillary or chromophobe subtype (Table 1).

It has been reported that, up to 26% of the patients with renal masses have preexisting stage 3 and higher chronic kidney disease (CKD), defined as a glomerular filtration rate (GFR) of <60 mL/min/1.73 m^2^ [4]. Additionally, radical nephrectomy has been regarded as a significant risk factor for developing CKD [4]. Median preoperative eGFR was 89.37 mL/min/1.73 m^2^ (range = 60–136) in our cohort, which indicates stage 2 CKD. This mild renal impairment in our study population with a mean age of 50 years and mean ASA score of 1.18 should be interpreted cautiously. Our cohort may be subjected to selection biases and therefore may not reflect the actual renal functional status of the patients awaiting nephron-sparing surgery. In other clinical series dealing with NSS for T2 masses, median preoperative eGFR varied between 65 and 92 mL/min/1.73 m^2^. In an era, where the incidence of metabolic syndrome and associated clinical consequences are on the rise [29], these facts should be considered before planning surgery for any renal mass, not only for T1 lesions or imperative indications. Furthermore, renal functional status should be evaluated by eGFR calculations, since serum creatinine value is not a sensitive tool that can detect early phases of renal impairment. The importance of eGFR was emphasized by a study showing that as many as 12.5% of all patients and 23% of patients aged ≥70 years presenting with an enhancing renal mass and normal serum creatinine (<1.4 mg/dL) had unrecognized CKD stage 3 or higher [30].

In the clinical series evaluating the results of NSS for tumors >7 cm in size (Table 2), renal functional maintenance, which may be considered as the primary advantage of NSS, has been highlighted. Long et al. reported advancement in CKD stage in only 11% of their study population [17]. Breau et al. detected a median increase in serum creatinine value by 9.5% compared to 33% for RN [20]. In the studies conducted by Becker et al. [18] and Karellas et al. [19], median eGFR decreased after NSS. Peycelon et al. demonstrated a small increase in mean serum creatinine values at followup in their small cohort of patients [22]. In our study, median eGFR value decreased from 89.37 to 79.15 mL/min/1.73 m^2^ postoperatively and CKD stage progressed in 5 patients (29.4%). Temporary renal replacement therapy, in the form of hemodialysis, had to be initiated for one patient who had a large (9 cm) upper pole mass in his solitary kidney. Although the surgery was carried under nonischemic conditions, the extent of the resection may be the factor underlying the need for renal replacement.

Our study has several limitations such as the retrospective nature with its inherent biases, small sample size, heterogeneous study population, limited follow-up duration, and pathological understaging in 5 patients. Nonetheless, our findings are in concordance with the previous studies, encouraging the utility of NSS for renal masses larger than 7 cm.

## 5. Conclusions

Nephron-sparing surgery is a feasible option for renal masses, measuring larger than 7 cm on preoperative imaging studies. Despite a limited duration of followup in our series, oncologic results are promising. Renal functional status did not change significantly after NSS for large (>7 cm) renal masses. Less than 1/5 of the study population suffered grade 2 and higher complications during the postoperative period. Regardless of tumor stage, nephron-sparing surgery may be considered for carefully selected patients, including those with a normally functioning contralateral kidney.

## Figures and Tables

**Table 1:** Histopathological profile of the renal masses, with a maximal diameter of more than 7 cm on preoperative imaging, which were managed by NSS.

NSS for renal masses >7 cm on preoperative imaging (*n* = 17)						
Histology (*n*, %)				Grade (*n*, %)		
RCC (13, 76.47%)		Benign (4, 23.52%)		Fuhrman-1	Fuhrman-2	Fuhrman-3
Clear cell	8	Angiomyolipoma	3			
Papillary	2	Cystic nephroma	1	2, 11.76%	8, 47.05%	3, 17.64%
Chromophobe	3					

**Table 2:** Outcomes of the case series dealing with nephron-sparing surgery for renal masses measuring more than 7 cm.

Reference number	Number of tumors ≥7 cm	Median followup (months)	Positive margins (*n*, %)	Overall survival at 5 years (%)	Median tumor size (cm)	Median preoperative creatinine or eGFR	Median postoperative creatinine or eGFR
[17]	49	13.1	5 (10.2)	94.5	8.7	1.18 mg/dL	1.30 mg/dL
[18]	91	28	NR	88	8.0	92.1 mL/min	81 mL/min
[19]	37	17	0	NR	7.5	65 mL/min	55 mL/min
[20]	57	38	NR	75	7.5	1.2 mg/dL	9.5% increase
[21]	29	54	NR	84	8.5	NR	NR
[22]	16	70	5 (31)	66	8.4	1.32 mg/dL	1.41 mg/dL
[23]	50	47	NR	82	9.9	NR	NR
Present study	17	35.2	0	NR	8.24	89.3 mL/min	76.9 mL/min

NR: not reported; eGFR: estimated glomerular filtration rate.
